# Le syndrome de Budd-Chiari: une complication rare de la sarcoïdose hépatique (à propos d'un cas)

**DOI:** 10.11604/pamj.2016.23.4.8564

**Published:** 2016-01-17

**Authors:** Ismael Ait Sghier, Nabil Moatassim Billah

**Affiliations:** 1 Service de Radiologie Centrale, Hôpital Avicenne, Rabat, Maroc

**Keywords:** Sarcoïdose, syndrome de Budd-Chiair, granulomatose, Sarcoidosis, Budd-Chiari syndrome, granulomatosis

## Abstract

L'atteinte hépatique au cours de la sarcoïdose est une localisation fréquente, habituellement asymptomatique. La cholestase anictérique et l'hypertension portale représentent ses principales complications. Le syndrome de Budd-Chiari est une complication peu connue qui demeure exceptionnelle. Nous rapportons un nouveau cas de syndrome de Budd-Chiari compliquant une sarcoïdose hépatique chez une jeune femme de 45 ans.

## Abstract (en)

Sarcoidotic involvement of the liver is frequent, albeit uncommonly symptomatic. Anicteric cholestasis and portal hypertension are the main complications. Budd-Chiari syndrome is a little known and exceptional complication of hepatic sarcoidosis. We present a case of A 45-year-old woman suffering from hepatic sarcoidosis who developed Budd-Chiari syndrome.

## Introduction

L'atteinte hépatique représente une localisation bien connue de la sarcoïdose; elle n'est symptomatique que dans le tiers des cas. Ces complications sont rares, représentées essentiellement par la cholestase anictérique et l'hypertension portale. Le syndrome de Budd-Chiari est exceptionnel. Nous rapportons un nouveau cas de syndrome de Budd-Chiari compliquant une sarcoïdose hépatique chez une jeune femme de 45 ans.

## Patient et observation

Une patiente de 45 ans, suivie pour sarcoïdose avec localisation pulmonaire, était admise pour bilan étiologique d'une hépatomégalie de découverte fortuite. L'examen clinique était sans particularité en dehors d'une hépatomégalie à 22 cm. Le bilan biologique avait conclu à une cholestase anictérique sans cytolyse ni syndrome inflammatoire, et avec un dosage de l'enzyme de conversion de l'angiotensine à 3 fois la normale.

**Figure 1:**
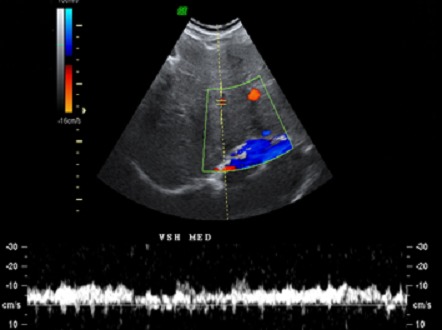
Échographie doppler hépatique: aspect grêle de la veine hépatique médiane qui garde un flux multiphasique avec absence d'identifications des veines hépatiques gauche et droite

L’échographie abdominale mettait en évidence un foie dysmorphique, augmenté de taille, d’échostructure hétérogène avec hypertrophie du segment I et de multiples adénopathies hilaires hépatiques et coelio-mésentériques. L’étude doppler montrait une perméabilité partielle de la veine hépatique médiane, qui présentait un aspect grêle et un flux multiphasique, avec sténose des veines hépatiques droite et gauche La veine cave était perméable, sans anomalie morphologique ou hémodynamique (Figure 1).

**Figure 2:**
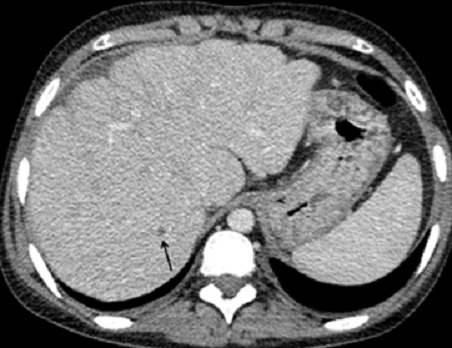
Coupe TDM axiale avec injection de produit de contraste: foie dysmorphique de contours lobulés avec hypertrophie du segment I; noter la présence d'un nodule hypodense infracentimétrique non rehaussé après injection de produit de contraste (flèche) - nodule sarcoïdosique-ainsi qu'une lamé d’épanchement péritonéal préhépatique

**Figure 3:**
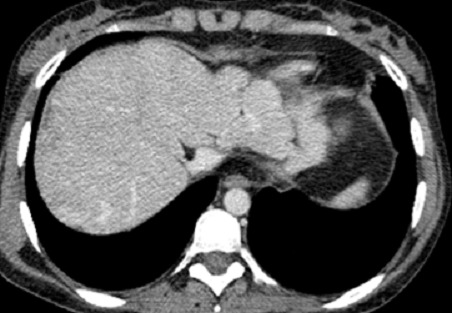
Absence d'opacification des veines hépatiques avec bonne opacification de la veine cave inférieure

**Figure 4:**
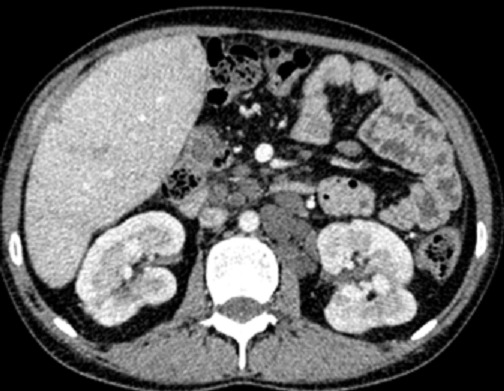
Coupe TDM axiale avec injection de produit de contraste montrant de multiples micronodules hépatiques avec des adénopathies coelio-mésentériques et latéro-aortiques

La tomodensitométrie thoraco-abdominale confirmait les données de l’échographie-Doppler abdominale avec mise en évidence quelques nodules hypodensesinfracentimétriques non rehaussés après injection de produit de contraste évoquant des nodules sarcoïdosiques (Figure 2, Figure 3 et Figure 4), ainsi que de multiples adénomégalies intra-abdominales (Figure 4). L’étage thoracique était sans anomalie. L'examen histologique hépatique avait conclu à la présence de granulomes épithélioïdes et gigantocellulaires sans nécrose caséeuse. Le bilan tuberculeux, immunologique et la recherche d'une thrombophilie, étaient négatifs. Le diagnostic d'un syndrome de Budd-Chiari compliquant une sarcoïdose hépatique était ainsi retenu.

## Discussion

La sarcoïdose est une maladie systémique d’étiologie inconnue, caractérisée par la présence de lésions granulomateuses non caséeuses dans les organes atteints [1]. Bien que l'atteinte médiastino-pulmonaire soit de loin la plus fréquente (90%), tous les organes peuvent en effet être atteints. L'atteinte hépatique représente une localisation fréquente, retrouvée dans 50 à 80% des biopsies hépatiques en cas de sarcoïdose systémique [2]. Elle est dans la majorité des cas asymptomatique [3]. Le signe le plus souvent retrouvé en imagerie est une hépatomégalie. Elle est retrouvée à l’échographie ou au scanner abdominal dans plus de 50% des cas [4]; suivie des nodules hépatiques, qui ne sont observés que dans 5% des cas [4].

Les complications de la sarcoïdose hépatique sont rares. Les deux principales complications sont la cholestase chronique et l'hypertension portale qui ne sont rencontrées que dans moins de 5% des cas. Le syndrome de Budd-Chiari reste une complication exceptionnelle. Celui-ci serait la conséquence d'une compression extrinsèque des veines hépatiques par des granulomes sarcoïdosiques ou d'une infiltration de la paroi des veines hépatiques entrainant une stase veineuse favorisant la formation de thrombus [5]. Le tableau clinique dépend du nombre de veines obstruées et de la rapidité d'installation de la thrombose. Les formes aiguës peuvent se révéler par une insuffisance hépatique, des douleurs abdominales, une fièvre et/ou une ascite exsudative. Mais le plus souvent, la découverte est tardive, ce qui pose le problème du diagnostic différentiel avec la cirrhose. Les formes chroniques sont en rapport avec une obstruction progressive, et sont le plus souvent pauci- ou asymptomatiques. Ce fut le cas chez notre patiente qui ne présentait cliniquement aucun signe, en dehors d'une hépatomégalie. A ce stade, l'exploration radiologique retrouve un foie hétérogène, dysmorphique avec hypertrophie du segment I, des signes d'HTP avec une circulation collatérale, une ascite et des anomalies de perfusion du parenchyme hépatique, mieux étudiés sur l'imagerie en coupe (TDM ou IRM).

Le développement des nodules de régénération a été décrit à ce stade également. Ils sont la conséquence des modifications de la perfusion hépatique (diminution du flux portal avec hyperartérialisation hépatique). Ils sont typiquement multiples, le plus souvent inférieures à 3 cm, homogènes, hypo ou hyperéchogènes à l’échographie, iso ou hyperdenses au scanner, hyperintense en T1, iso ou hypointense en T2 à l'IRM avec prise de contraste intense au temps artériel. Ces nodules ne doivent pas être confondus avec les nodules sarcoïdosique qui résultent de la confluence des granulomes. Comme les nodules de régénération, ces derniers peuvent être extrêmement nombreux, de taille variable mesurant le plus souvent moins de 2 cm de diamètre. Ils sont hypoéchogènes (moins souvent hyperéchogènes) à l’échographie, hypodenses au scanner [6], hypo-intense sur toutes les séquences en IRM et restent non rehaussés après l'injection de produit de contraste. Ces nodules sont exceptionnellement calcifiés.

Le traitement du syndrome de Budd-Chiari est fondé sur le contrôle de la cause, sur la prévention des thromboses veineuses et sur le rétablissement du drainage veineux hépatique. En cas d'insuffisance hépatocellulaire rapidement progressive, la transplantation hépatique doit être discutée.

## Conclusion

L'atteinte hépatique au cours de la sarcoïdose est une localisation fréquente, habituellement asymptomatique. La dissémination des granulomes dans le parenchyme hépatique peut se compliquer d'une cholestase anictérique, d'hypertension portale et exceptionnellement d'un syndrome de Budd-Chiari. Malgré l'exceptionnelle association du syndrome de Budd-Chiari et de la sarcoïdose hépatique, ce diagnostic doit toujours être évoqué devant le moindre signe d'appel, justifiant ainsi la réalisation d'une échographie-doppler hépatique au moindre doute -voire chez des patients asymptomatiques- afin d’établir le diagnostic à un stade précoce.
